# Placebo prescription and empathy of the physician: A cross-sectional study

**DOI:** 10.1080/13814788.2017.1291625

**Published:** 2017-03-28

**Authors:** João Braga-Simões, Patrício Soares Costa, John Yaphe

**Affiliations:** ^a^ Life and Health Sciences Research Institute (ICVS), School of Medicine, University of MinhoBragaPortugal; ^b^ Alto Minho Local Health Unit EPEViana do CasteloPortugal; ^c^ ICVS/3B’s—PT Government Associate LaboratoryBraga/GuimarãesPortugal; ^d^ Faculty of Psychology and Educational Sciences, University of PortoPortugal

**Keywords:** Placebo prescription, physician empathy, general practice

## Abstract

**Background:** Empathy in the patient–physician relationship is a major component in an effective placebo treatment, as in every medical treatment. Understanding the role of empathy of the physician in the placebo effect may help dissect some of the context variables responsible for the effectiveness of the placebo.

**Objectives:** To determine the frequency of placebo prescription, doctors’ beliefs, motivation, and attitudes to placebos in general practice in northern Portugal and to test the association between placebo prescription and physician empathy.

**Methods:** A cross-sectional study was conducted between November 2014 and January 2015 among general practice specialists and interns from 14 health centres in a northern Portuguese health region. The self-report questionnaire included the Portuguese version of the Jefferson scale of physician empathy (JSPE) and a questionnaire about placebo prescription. Associations between demographic variables, JSPE score, prescription of placebo, and the attitudes to placebo score were tested with the chi-squared statistic, student *t*-tests for independent samples, and Pearson correlation.

**Results:** The study included 93 general practitioners (GP) (response rate: 74%). Placebos were prescribed by 73% (*n* = 68) of the respondents. GPs who prescribe placebo are significantly younger (mean age = 38.4 years; SD = 11.1; *t* (90) = 2.98, *P* <.05, d = 0.67) than non-prescribers (mean age =46.5 years; SD =13.3). Favourable attitudes towards placebo prescription are associated with higher empathy scores (R = 0.310, *P* <.01).

**Conclusion:** Placebo prescription is frequent and associated with empathy from the prescriber, especially among younger GPs.

KEY MESSAGES

- Placebo prescription is frequent in northern Portugal, especially among younger GPs.
- GPs with higher empathy scores are more favourable to placebo prescription.

## Introduction

The effectiveness of most medical interventions partially relies on contextual or subjective factors, aside from the tangible and objective treatment methods already established and used in medicine [1]. Thus, empathy, as a critical factor in the clinical context, has been found to positively affect outcomes of both placebo treatments and bioactive treatments [2]. It has become clear that placebo responses derive not only from the belief that a bioactive compound was administered or that a legitimate medical procedure was undergone but also from environmental and psychosocial factors embedded within clinical and research encounters [3]. Nonetheless, most placebo research has been focused on understanding the underlying neurobiological mechanisms of the patient’s response to placebos, and less on understanding the physician component of the clinical dyad [4].

Recent evidence suggests that medical providers use placebo treatments widely, and even that patients can be open for those interventions under certain circumstances [5–7]. Among primary healthcare, although the extent of this practice is highly variable, international retrospective studies report figures ranging from 17% to 99% of general practitioners (GP) using placebo treatments at least once in their career [5]. The distinctive features of general practice, where clinicians see patients with a wide variety of complaints and symptoms, serving unselected populations, with diagnostic uncertainty, pressure to treat, and time constraints, make placebo prescription a frequent option [5].

As a psychobiological process, the placebo effect is due to the therapeutic context, including the patient, the physician, and their interaction with the surrounding environment [8]. Previous research stresses the importance of the healing context for therapeutic effect and the placebo effect. Both rely on expectations, conceptions, and prejudices as factors that promote or compromise the healing process [9]. In one representative study of irritable bowel syndrome, the positive effects of placebo acupuncture were enhanced if promoted by an empathic physician [2]. This emphasizes the role of empathy as a major component of the doctor–patient relationship and the healing process, contributing as an important factor to the placebo effect.

Despite the potential role of empathy, there are no studies exploring the relationship between physician empathy and attitudes, beliefs, and practices regarding placebo prescription. The objectives of this study were to determine the extent of placebo prescription by GPs in one region and test the associations between placebo prescription and physician empathy.

## Methods

### Study design

The study population consisted of specialist GPs and second, third and fourth-year GP interns in the 14 health centres of Matosinhos local health unit (MLHU), an urban area in northern Portugal. First-year GP interns were excluded from the study because they are not licensed to prescribe medication. One of the 14 health centres failed to reply to the request for participation and was excluded from the study. Questionnaires on placebo prescription were distributed in the health centres between November 2014 and January 2015.

### Ethics

Ethical approval for this study was obtained from the University of Minho Life Sciences Ethics Committee (Document SECVS 145/2014) and the MLHU Ethics Committee (reference 079/CE/JAS).

### Questionnaires

A self-reported anonymous questionnaire composed of two sections was used. The first section assessed the frequency and circumstances of placebo prescription, providing a working definition of placebos to standardize interpretations adapted from other international studies on this subject [10–13]. The questionnaire is provided in Supplementary material (available online). The second section consisted of the validated Portuguese version of the Jefferson scale of physician empathy (JSPE) [14,15]. The (JSPE) is an instrument developed to measure empathy in physicians and medical students and has been found to be valid in some settings [15].

To assess the time required for completion of the forms, face validity, and comprehension, the questionnaire was tested in a pilot study with six GPs from the target population who were excluded from the final study.

### Statistical analysis

Statistical analyses were conducted considering the variables measurement level. Assumptions for all tests were considered. To test the normality of the distribution of scale variables, Kolmogorov–Smirnov (KS) tests were conducted. When KS tests were statistically significant, we used the following rules-of-thumb: absolute skewness and kurtosis values lower than 3.0 and 8.0, respectively, and results did not show significant diversion from a normal distribution [16]. Group differences were tested through chi-square independence tests and student *t*-tests for independent samples depending on variables measurement level. The strength of association between two scale variables was determined by Pearson correlation coefficients. An exploratory factor analysis was conducted with the questionnaire items evaluating attitudes towards placebo use. Internal consistency of the dimension of attitudes towards placebo prescription and of the JSPE subscales and total score were determined by Cronbach’s alpha. Statistical analyses were conducted using IBM-SPSS Statistics^®^ (version 22). *P*-values <.05 were considered significant and effect sizes were interpreted according to values given by Cohen (1988) [17].

## Results

### Sample

From the 126 questionnaires initially distributed, a gender and age representative sample of 93 GPs (response rate: 74%) was obtained. Table 1 presents the demographics of the study sample. The mean age of participants was 41 years, with a mean of 14 years’ seniority and 80% of the physicians were female.

**Table 1.** Demographic characteristics of general practitioners included in the study.

Age (years; mean ± SD)	41 ± 12
Female (*n*)	80% (74)
Seniority (*n*)	
GP specialist	77% (72)
General practice intern	23% (21)
Years of clinical practice (mean ± SD)	16 ± 12

SD, standard deviation.

### Placebo prescription

Of the surveyed GPs, 73% (*n* = 68) prescribed placebos. When asked about the frequency with which they prescribed placebos, 43% (*n* = 29) reported doing so several times a year, 34% several times a month, 10% (*n* = 7) several times a week, and 3% (*n* = 2) prescribed placebos several times a day. Ten per cent (*n* = 7) of the GPs did not know how often they prescribed placebos or ignored the question.

Associations between demographic variables and placebo prescription were tested (Table 2). A significant association was found between age and placebo prescription. Younger GPs (mean age = 38.4 years; SD = 11.1) were more likely to be prescribers than older GPs who were non-prescribers (mean age = 46.5 years; DP = 13.3), *t* (90) = 2.98, *P* ≤.05, two-tailed, d = 0.67). GPs with fewer years of clinical practice (mean seniority = 13.8 years; SD = 11.4) were more likely to be placebo prescribers, than more experienced GPs (mean seniority = 20.4 years; SD = 13.9; *t* (36.4) = 2.13, *P* ≤ .05, two-tailed, d = 0.51). The effect sizes (Cohen’s d = 0.67 and 0.51, respectively) suggest a moderate to high practical significance of the results. No associations were found between gender and placebo prescription.

**Table 2.** Relations between placebo prescription and sociodemographic characteristics.

	Prescribes placebo			
	Yes	No	*P*	Effect size
Gender (*n*)				
Female	73% (54)	27% (30)	>.2	Φ = –0.006
Male	73% (14)	26% (5)		
Seniority (*n*)				
GP Specialist	72% (52)	27% (20)	>.2	Φ = –0.037
GP Intern	76% (16)	23% (5)		
Age (years; mean ± SD)	38.4 ± 11.1	46.5 ± 13.3	<.01	d = 0.67
Years of clinical practice (mean ± SD)	13.8 ± 11.4	20.4 ± 13.9	<.05	d = 0.51

GP, general practitioner; SD, standard deviation.

### Patient information

The ways GPs inform patients about placebo prescription varied. Many GPs, 43% (*n* = 29) say nothing to the patient about this, 34% (*n* = 23) stated that the prescription is a medicine without specific effects on the patient’s ailment, 3% (*n* = 2) state that the prescription is a medicine, and 10% (*n* = 7) state directly to the patient that the prescription is a placebo.

### Motivations for placebo prescription

The motivations reported by the GPs to justify placebo prescription are shown in Table 3. The options ‘to calm the patient’ (60%, *n* = 41), and ‘as a diagnostic tool (to distinguish between real and imaginary symptoms or organic and psychological symptoms)’ (60%, *n* = 41), were the most common justifications given for placebo prescription.

**Table 3.** Motivations for placebo prescription among northern Portuguese GPs (*n* = 68).

In which situations would/did you prescribe a placebo?	(*n*)
As a diagnostic tool (to distinguish between real and imaginary symptoms, or organic and psychological symptoms)	60% (41)
To calm a patient	60% (41)
As a treatment for an unspecific symptom	47% (32)
To appease a complaining patient	46% (31)
As a substitute for a medicine without rationale but the patient expected it	38% (26)
As a supplement for other medicine	37% (25)
As a substitute while titrating the dose of a medicine (e.g. psychotropic medication withdrawal)	21% (14)
For pain control	15% (10)

### Perception of placebo efficacy

Many GPs, 43% (*n* = 29), stated that the placebos prescribed are ‘frequently’ effective, 34% (*n* = 23) stated placebos are ‘sometimes’ effective, 10% (*n* = 7) considered that placebos are ‘never’effective and, 3% (*n* = 2) claimed to ignore the efficacy of the placebos prescribed.

### Attitudes towards placebo prescription

The participants were asked to provide a semi-quantitative evaluation of agreement with seven statements about ethical and professional components of placebo prescription on a seven-item Likert-type scale (Table 4). Most GPs believe their patients could benefit from placebos and are favourable to the prescription of placebos as a therapeutic tool. Clinical practice and scientific evidence of placebo effectiveness are important factors to consider when prescribing placebos. The item that most divided opinions among GPs was the need for prior informed consent. The results were concordant when responses to statements regarding the ethics of placebo prescription and prohibition of placebo prescription were compared. Most GPs who disagreed with the statement that placebo prescription is unethical also disagreed with the statement that placebo prescription should be prohibited.

**Table 4.** Attitudes and ethical considerations towards placebo prescription. Results of the seven-item Likert scale survey assessing attitudes towards placebo prescription.

Regarding placebo prescription, how much do you agree/disagree with the following statements?	Score (mean ± SD)	Agreement^a^
My position about placebo prescription is that it should be:		
Always forbidden.	2.4 ± 1.4	7%
Allowed if scientific data of efficacy exists.	5.0 ± 1.6	66%
Allowed if my clinical experience supports efficacy.	4.4 ± 1.6	54%
Allowed after informed consent.	4.0 ± 2.0	40%
I consider that my patients could benefit from placebos.	4.7 ± 1.5	60%
I consider that placebo prescription could be included in the therapeutic arsenal.	4.6 ± 1.6	58%
I consider placebo prescription ethically reprehensible.	2.3 ± 1.3	7%

^a^ Cumulative frequencies of Likert scale 4 to 7.

SD, standard deviation.

Exploratory factor analysis (EFA) with principal components method of factor extraction was conducted with the items evaluating attitudes towards placebo prescription (Supplementary material: Table S1). Items 6a and 6d (‘allowed if clinical practice corroborates efficacy’ and ‘allowed after informed consent’) were excluded for low communality (<0.5) and a new EFA was conducted, revealing a unifactorial structure, named score of attitudes towards placebo prescription (SATPP, Cronbach’s alpha = 0.751). Results from tests of item saturation were satisfying, as well as internal consistency [18].

### Jefferson scale of physician empathy

From the 93 questionnaires, only 88 JSPE questionnaires were correctly completed. Total scores of the JSPE showed a good internal consistency (20 items, Cronbach’s alpha = 0.804). The three dimensions of the JSPE, perspective taking (PT, 10 items), empathic care (EC, 7 items) and, standing in patient’s shoes (SPS, three items) showed acceptable internal consistency (PT, α = 0.655, item 18 excluded) and good internal consistency (EC, α = 0.780; SPS, α = 0.718) [18].

Cross-tabulations (Table 5) reveal that higher scores on the empathic care dimension of JSPE are significantly associated with placebo prescribers (M = 64, SD = 6, *t* (86) = 2.038, *P* = .045) than non-prescribers (M = 61, SD = 9). More favourable attitudes towards placebo prescription, measured by SATPP, are significantly associated with higher JSPE total scores (Pearson’s r (87) = 0.310, *P* = .003) and in perspective taking (Pearson’s r (88) = 0.219, *P* = .041) and empathic care (Pearson’s r (88) = .293, *P* = .006). Being a GP intern is also associated with higher scores of empathic care (M = 65, SD = 5, *t* (86) = −1.983, *P* = .052) than senior GPs (M = 63, SD = 8). No significant association was found between gender, age and JSPE scores and dimensions.

**Table 5.** Associations between empathy (JSPE) results, placebo prescription and demographic characteristics

	Total JSPE score(mean ± SD)	Perspective taking(mean ± SD)	Empathic care(mean ± SD)	Standing in patient’s shoes(mean ± SD)
Prescribes placebo				
*Yes*	120 ± 11	38 ± 4	64 ± 6	17 ± 3
*No*	115 ± 15	38 ± 5	61 ± 9	16 ± 4
*P*	0.111	0.788	0.045	0.356
*D*	0.380	0	0.392	0.283
SATPP				
*P*	0.003	0.041	0.006	0.085
*R*	0.310	0.219	0.293	0.186
Status				
* *Specialist GP	118 ± 13	38 ± 5	63 ± 8	17 ± 3
* *GP intern	121 ± 8	39 ± 3	65 ± 5	16 ± 3
*P*	0.329	0.413	0.052	0.365
*D*	–0.278	–0.243	–0.300	0.333
Gender				
* *Female	117 ± 13	38 ± 3	63 ± 7	17 ± 3
* *Male	119 ± 12	38 ± 5	62 ± 8	17 ± 4
*P*	0.563	0.582	0.564	0.942
*D*	–0.160	0	0.133	0
Age				
*P*	0.563	0.325	0.202	0.533
*R*	–0.063	0.107	–0.138	–0.068

JSPE, Jefferson scale of physician empathy; SATPP, score of attitudes towards placebo prescription.

Measures of effect size: r = Pearson correlation Coefficient; D = Cohen’s d.

## Discussion

### Main findings

This study among 93 Portuguese GPs provides the first evidence for the extent of placebo prescription in general practice in Portugal. Seventy-three per cent of them admit prescribing placebos with varying frequency. The circumstances of placebo prescription varied. It is prescribed most often as a diagnostic tool to distinguish real from imaginary symptoms, to appease the patient, and as a treatment for nonspecific symptoms. The perception of the efficacy of placebos is high and many GPs do not explain to the patient that they are prescribing a placebo.

### Strengths and limitations

The definition of placebo and measures of frequency of prescription vary significantly between studies making comparisons difficult. However, it is evident that placebo prescription in northern Portugal is frequent, particularly among younger GPs and interns. In the only study where (pure) placebo prescription was evaluated among interns (in internal medicine), 16% of the respondents claimed to have already prescribed placebos, while in the current study 76% of GP interns claim to have done so [19]. This difference may be due to the inclusion of impure placebos in the definition of placebos in the current study and the fact that the two studies assessed different medical specialties.

This study enrolled 126 GPs and interns from 14 health centres, representative of one Portuguese health region, and obtained a good response rate. Even though this may not be representative of all Portuguese GPs and the findings may reflect selection bias, it provides a first clear look at placebo prescription in Portugal.

There are no validated questionnaires in Portuguese regarding placebo prescription and circumstances of placebo prescription in clinical practice. The questionnaire used could benefit from further validation.

### Comparison with existing literature

The main results of this study are consistent with previously published findings, where the proportion of placebo prescription ranged from 17% to 99% of GPs [5,13]. Thirty-four per cent of GPs in this study claim to prescribe placebos several times a month and, 43% claim to prescribe them several times a year. In other studies, wide variability in frequency was noted. For example, 45% of German GPs prescribe placebos at least once per year, and 38% of Danish GPs prescribe 1 to 10 times per year [13,20].

Placebo prescription is a controversial subject, which required this study to address the ethical and professional issues involved in a broad manner (Table 4). Although most GPs in this study were against the prohibition of placebos (75%) and do not consider it ethically wrong (81%), there were still 25% of GPs who disagree with the prescription of placebos in medical care. Furthermore, from the 27% of GPs who do not prescribe placebos, only 7% feel this should be prohibited. The reasons for this behaviour are probably linked to deontological constraints involved in the act of prescribing a placebo given that there is an ethical and legal obligation for telling the truth to patients, and because the use of placebo is only legally sanctioned in research when subjects give informed consent [21].

There were no published studies found testing the associations between empathy and placebo prescription. In this study, we demonstrated a significant association between placebo prescription and higher scores of empathy measured by the JSPE. Moreover, favourable attitudes towards placebo prescription, summarized in the attitudes score we generated (the SATPP), were associated with higher scores on the JSPE and two of its dimensions, empathic care and perspective taking.

In some studies, a decline in empathy was observed among medical students during their training [22]. Attitudes and beliefs toward placebo prescription may also change during medical education [23]. However, we cannot elaborate if changes in empathy are associated with changes in attitudes towards placebo prescription over time. This might be clarified in longitudinal studies.

### Implications for clinical practice and research

Since empathy is a crucial element in the healing context, this study was designed to test the association between placebo prescription and empathy of the prescribing physician. These findings may help us understand why placebos are effective and what are the context conditions responsible for its effect, dissecting the science of placebo and enabling the physician to harness the contextual factors and the conditions that promote a beneficial healing environment.

Due to ethical and professional constraints, the exact extent of placebo prescription is difficult to assess and will remain controversial in the medical community and among the public. Prospective studies with the debriefing of physicians to understand their prescribing choices may contribute to clarify this issue further.

The results from this study suggest that education of medical students and interns is necessary to raise awareness about placebos and in forming opinions about their ethical use. The inclusion of the topic of placebo prescription in the medicine schools’ curricula may improve decision making of future doctors when facing this subject.

## Conclusion

Placebo prescription is frequent among GPs in northern Portugal and most frequent among younger GPs. Empathy of the GPs is associated with openness to placebo prescription. Our results come from a narrow geographic area and may need to be repeated in other regions. However, it seems clear that a wider debate about placebo prescription in clinical practice, including therapeutic, legal and ethical aspects is required.

## Supplementary Material

Placebo Prescription in General Practice - Questionnaire

Supplementary Material: Table S1

## References

[1] Kisaalita N, Staud R, Hurley R, et al Placebo use in pain management: the role of medical context, treatment efficacy, and deception in determining placebo acceptability. Pain. 2014;155:2638–2645. 25267208 10.1016/j.pain.2014.09.029 PMC4250369

[2] Kaptchuk TJ, Kelley JM, Conboy LA, et al Components of placebo effect: randomised controlled trial in patients with irritable bowel syndrome. BMJ. 2008;336:999–1003. 18390493 10.1136/bmj.39524.439618.25 PMC2364862

[3] Fontaine KR, Williams MS, Hoenemeyer TW, et al Placebo effects in obesity research. Obesity (Silver Spring). 2016;24:769–771. 27028278 10.1002/oby.21456 PMC4817362

[4] Decety J, Fotopoulou A. Why empathy has a beneficial impact on others in medicine: unifying theories. Front Behav Neurosci. 2015;8:457. 25642175 10.3389/fnbeh.2014.00457 PMC4294163

[5] Fässler M, Meissner K, Schneider A, et al Frequency and circumstances of placebo use in clinical practice-a systematic review of empirical studies. BMC Med. 2010;8:15. 20178561 10.1186/1741-7015-8-15 PMC2837612

[6] Kaptchuk TJ, Friedlander E, Kelley JM, et al Placebos without deception: a randomized controlled trial in irritable bowel syndrome. PloS One. 2010;5:e15591. 21203519 10.1371/journal.pone.0015591 PMC3008733

[7] Hull SC, Colloca L, Avins A, et al Patients’ attitudes about the use of placebo treatments: telephone survey. BMJ. 2013;347:f3757. 23819963 10.1136/bmj.f3757 PMC3698941

[8] Linde K, Fässler M, Meissner K. Placebo interventions, placebo effects and clinical practice. Philos Trans R Soc Lond B Biol Sci. 2011;366:1905–1912. 21576148 10.1098/rstb.2010.0383 PMC3130396

[9] Di Blasi Z, Harkness E, Ernst E, et al Influence of context effects on health outcomes: a systematic review. Lancet. 2001;357:757–762. 11253970 10.1016/s0140-6736(00)04169-6

[10] Fässler M, Gnädinger M, Rosemann T, et al Use of placebo interventions among Swiss primary care providers. BMC Health Serv Res. 2009;9:144. 19664267 10.1186/1472-6963-9-144 PMC2731747

[11] Fässler M, Gnädinger M, Rosemann T, et al Placebo interventions in practice: a questionnaire survey on the attitudes of patients and physicians. Br J Gen Pract. 2011;61:101–107. 21276337 10.3399/bjgp11X556209 PMC3026149

[12] Nitzan U, Lichtenberg P. Questionnaire survey on use of placebo. BMJ. 2004;329:944–946. 15377572 10.1136/bmj.38236.646678.55 PMC524103

[13] Meissner K, Höfner L, Fässler M, et al Widespread use of pure and impure placebo interventions by GPs in Germany. Fam Pract. 2012;29:79–85. 21808072 10.1093/fampra/cmr045

[14] Aguiar P, Salgueira A, Frada T, et al Physician Empathy: translation, validation and application of a scale. In: Proceedings of the X Galician-Portuguese Psychopedagogy International Congress. Braga: Minho University; 2009.

[15] Hojat M, Gonnella JS, Nasca TJ, et al The Jefferson scale of physician empathy: further psychometric data and differences by gender and specialty at item level. Acad Med. 2002;77:S58–S60. 12377706 10.1097/00001888-200210001-00019

[16] Kline R. Principles and practice of structural equations modeling. 2nd ed London: Guilford Press; 2005.

[17] Cohen J. Statistical power analysis for the behavioral sciences. 2nd ed Hillsdale, NJ: Lawrence Earlbaum Associates; 1988.

[18] Hair JF, Tathan RL, Anderson RE, et al Multi-variate data analysis. 5th ed Upper Saddle River, NJ: Prentice-Hall; 1998.

[19] Berger JT. Placebo medication use in patient care: a survey of medical interns. West J Med. 1999;170:93–96. 10063395 PMC1305448

[20] Hróbjartsson A, Norup M The use of placebo interventions in medical practice—a national questionnaire survey of Danish clinicians. Eval Health Prof. 2003;26:153–165. 12789709 10.1177/0163278703026002002

[21] Blease C, Colloca L, Kaptchuk TJ. Are open-label placebos ethical? Informed consent and ethical equivocations. Bioethics. 2016;30:407–414. 26840547 10.1111/bioe.12245 PMC4893896

[22] Neumann M, Edelhäuser F, Tauschel D, et al Empathy decline and its reasons: a systematic review of studies with medical students and residents. Acad Med. 2011;86:996–1009. 21670661 10.1097/ACM.0b013e318221e615

[23] Lim EC-H, Seet RCS. Attitudes of medical students to placebo therapy. Intern Med J. 2007;37:156–160. 17316333 10.1111/j.1445-5994.2007.01264.x

