# Supplementary material for: Placebo prescription and empathy of the physician: A cross-sectional study
Source: Eur J Gen Pract. 2017 Mar 28;23(1):98–104. doi: 10.1080/13814788.2017.1291625 (PMC5774274; doi:10.1080/13814788.2017.1291625)
Supplement: Placebo Prescription in General Practice - Questionnaire [file igen_a_1291625_sm3996.docx]

**Placebo Prescription in General Practice - Questionnaire**

This survey is part of study addressing placebo prescription in clinical context among General Practitioners. The questionnaire is of self-report and anonymous. The collected data will be used only with academic and scientific purposes.

**Estimated time to complete questionnaire: 5-6 minutes**

**Placebo**: substance or treatment used for its non-specific psychological or psycho-physiological effects without specific effects for the condition being treated. The definition includes a variety of medical interventions (*e.g.* inert substances or medicines, diagnostic techniques, etc.).

**Pure Placebo**: inert substances like physiological saline that may be used for diagnostic or beneficial therapeutic effects in situations where there is no evidence of benefit for their use.

**Impure Placebo**: substances with known pharmacological activity (like Vitamin B12) that may be used in situations where they have no known evidence for effectiveness.

Mark with a **X** the chosen answer

1. In my clinical practice I prescribe placebos

Yes __ No __ Don’t know/ Don’t answer __

(If **Don’t know/ Don’t answer** please follow to **item 6**)

1. I prescribe placebos:
   1. Daily __
   2. Weekly__
   3. Monthly __
   4. Annually __
   5. Never __
   6. Don’t know/ Don’t answer __
2. Before prescribing placebos, I typically inform the patient it is:
   1. A medicine __
   2. A placebo __
   3. A medicine without specific effect to the patient’s complaint __
   4. I say nothing __
3. When I prescribe placebos, these are:
   1. Often effective __
   2. Sometimes effective __
   3. Never effective __
   4. I ignore their efficacy __
4. Of the following situations, please mark all those in which you would or have prescribed placebos:
5. As a diagnostic tool (to distinguish between real and imaginary symptoms, or organic and psychological symptoms)
6. As a supplement for other medicine;
7. As a substitute while tittering the dose of a medicine (e.g. psychotropic medication withdrawal);
8. To calm a patient;
9. To appease a complaining patient ;
10. As a diagnostic tool (to distinguish between real and imaginary symptoms, or organic and psychological symptoms);
11. As a treatment for an unspecific symptom**;**
12. For pain control;
13. Other situations:_________________________________________________________

**In a 1 to 7 scale, with 1 – Completely Disagree and 7 – Completely Agree, answer the following questions:**

1. My position about placebo prescription is that it should be:
2. **Always forbidden.**

| Completely Disagree | 1 | 2 | 3 | 4 | 5 | 6 | 7 | Completely Agree |
| --- | --- | --- | --- | --- | --- | --- | --- | --- |

1. **Allowed if scientific data of efficacy exists.**

| Completely Disagree | 1 | 2 | 3 | 4 | 5 | 6 | 7 | Completely Agree |
| --- | --- | --- | --- | --- | --- | --- | --- | --- |

1. **Allowed if my clinical experience supports efficacy.**

| Completely Disagree | 1 | 2 | 3 | 4 | 5 | 6 | 7 | Completely Agree |
| --- | --- | --- | --- | --- | --- | --- | --- | --- |

1. **Allowed after informed consent.**

| Completely Disagree | 1 | 2 | 3 | 4 | 5 | 6 | 7 | Completely Agree |
| --- | --- | --- | --- | --- | --- | --- | --- | --- |

1. I consider that my patients could benefit from placebos.

| Completely Disagree | 1 | 2 | 3 | 4 | 5 | 6 | 7 | Completely Agree |
| --- | --- | --- | --- | --- | --- | --- | --- | --- |

1. I consider that placebo prescription could be included in the therapeutic arsenal.

| Completely Disagree | 1 | 2 | 3 | 4 | 5 | 6 | 7 | Completely Agree |
| --- | --- | --- | --- | --- | --- | --- | --- | --- |

1. I consider placebo prescription ethically reprehensible.

| Completely Disagree | 1 | 2 | 3 | 4 | 5 | 6 | 7 | Completely Agree |
| --- | --- | --- | --- | --- | --- | --- | --- | --- |

| Age: | | | _____ years | | | | |
| --- | --- | --- | --- | --- | --- | --- | --- |
|  | | | | | | | |
| Gender | | | | M |  | F |  |
|  | | | | | | | |
| Graduation year | | | | |  | | |
|  | | | | | | | |
| Seniority | | | | | | | |
| Senior General Practioner | | | | | | |  |
| Trainee | 2º Year | | | | | |  |
|  | 3º Year | | | | | |  |
|  | 4º Year | | | | | |  |
|  | | | | | | | |
| Nationality | |  | | | | | |
