# Supplementary material for: Placebo prescription and empathy of the physician: A cross-sectional study
Source: Eur J Gen Pract. 2017 Mar 28;23(1):98–104. doi: 10.1080/13814788.2017.1291625 (PMC5774274; doi:10.1080/13814788.2017.1291625)
Supplement: Supplementary Material: Table S1 [file igen_a_1291625_sm3985.docx]

**Supplementary Information – S1**

| **Suppl. Table 1.** Results of Exploratory Factor Analysis of the items included in the Score of Attitudes Towards Placebo Prescription (SATPP) | | | |
| --- | --- | --- | --- |
| **Questionnaire items assessing attitudes towards placebo prescription** | | **Loadings** | **Cronbach’s Alpha if item deleted** |
| 6.) My position about placebo prescription is that it should be: | 6.a) Always forbidden | .518 | .726 |
|  | 6.c) Allowed if my clinical experience supports efficacy | .536 | .728 |
| 7.) I consider that my patients could benefit from placebos. | | .786 | .661 |
| 8.) I consider that placebo prescription could be included in the therapeutic arsenal. | | .747 | .670 |
| 9.) I consider placebo prescription ethically reprehensible. | | .491 | .739 |
